# Elevated plasma midkine and pleiotrophin levels in patients with systemic lupus erythematosus

**DOI:** 10.18632/oncotarget.13658

**Published:** 2016-11-26

**Authors:** Guo-Cui Wu, Hui Yuan, Hai-Feng Pan, Dong-Qing Ye

**Affiliations:** ^1^ Department of Epidemiology and Biostatistics, School of Public Health, Anhui Medical University, Hefei, Anhui, China; ^2^ Anhui Provincial Laboratory of Population Health and Major Disease Screening and Diagnosis, Hefei, Anhui, China; ^3^ Department of Preventive Medicine, Wannan Medical College, Wuhu, Anhui, China

**Keywords:** midkine, pleiotrophin, systemic lupus erythematosus, IL-17

## Abstract

Emerging evidence suggests that two heparin-binding growth factor, midkine and pleiotrophin are implicated in the pathogenesis of autoimmune diseases including SLE. To investigate the plasma midkine and pleiotrophin levels in SLE patients, as well as their correlation with major clinical parameters and interleukin-17 (IL-17) level in SLE, 83 SLE patients and 123 controls including 20 rheumatoid arthritis (RA) patients, 21 Sjögren's syndrome (SS) patients and 82 healthy controls (HCs) were recruited. Plasma midkine, pleiotrophin and IL-17 levels were detected by ELISA. Midkine and pleiotrophin levels were significantly higher in SLE, RA and SS patients compared with HCs (all *P* < 0.05). There were significantly lower midkine and pleiotrophin levels in SLE compared to SS (*P* < 0.05 and *P* < 0.01, respectively). No significant differences in midkine and pleiotrophin levels were found between SLE and RA (*P* = 0.240 and *P* = 0.074, respectively). Both plasma midkine and pleiotrophin levels were associated with rash and anti-SSA in SLE. In addition, both midkine and pleiotrophin levels were positively associated with IL-17 level in SLE (both *P* < 0.001). Area under curve (AUC) of the receiver operating characteristic (ROC) curve for midkine and pleiotrophin were 0.606 (0.527–0.681) and 0.605 (0.526–0.680) respectively. In conclusion, elevated plasma midkine and pleiotrophin levels and their associations with rash, anti-SSA and IL-17 in SLE patients suggest their involvement in this disease.

## INTRODUCTION

Systemic lupus erythematosus (SLE), a prototypic systemic autoimmune disease, is characterized by the production of multiple autoantibodies, formation of immune complexes, tissue inflammation in multiple organs and elevated levels of proinflammatory cytokines in peripheral blood [1]. These cytokines collectively play crucial roles in accelerating systemic inflammation, local tissue damage and immunoreactions [2]. Dysfunctions of immune regulation mechanisms, such as imbalance of pro-inflammatory cytokines and anti-inflammatory cytokines, contribute to the development of SLE [3].

The cytokine midkine, a heparin-binding growth factor, was first discovered as a molecule involved in embryonic development [4]. Previous studies have revealed that upregulation of midkine promoted tumor growth, survival, invasion and angiogenesis [5–7]. Moreover, midkine may also stimulate inflammatory responses [8], since it has been demonstrated to induce the migration of inflammatory cells [9–11] and suppresses the expansion of regulatory T cell (Treg) by blocking the development of tolerogenic dendritic cells (DCs) [12]. Recently, there is increasing evidence that midkine is implicated in autoimmune and inflammatory diseases, such as rheumatoid arthritis (RA) [13–15], multiple sclerosis (MS) [12, 16–18] and inflammatory bowel disease (IBD) [19, 20]. The serum midkine level could serve as a marker of disease activity in RA and an indicator of a poor prognosis [15]. Removal of midkine suppressed the animal model of MS, experimental autoimmune encephalomyelitis (EAE) due to expansion of the Treg cell population and decrease in the number of autoreactive Th1 and Th17 cells [16, 18]. In RA patients, serum midkine levels were positively correlated with IL-17 [21]. All these findings suggest that midkine may be implicated in the pathogenesis of autoimmune diseases. However, up to now, the role of midkine in SLE is largely unknown.

Midkine is the founding member of the family of heparin-binding growth factors, it is structurally unrelated to any other growth factor family known so far consisting of only one other member named pleiotrophin [8]. Human midkine and pleiotrophin have about 50% sequence identity [22]. Both midkine and pleiotrophin have diverse functions, such as angiogenesis, oncogenesis and inflammation. The high expression levels of midkine and pleiotrophin in many types of cancers make them excellent as biomarkers and therapeutic targets for cancer [23, 24]. Up to date, the evidence of pleiotrophin in autoimmune diseases is also very limited.

In the present study, to further explore the role of midkine and pleiotrophin in human SLE, we investigated the plasma midkine and pleiotrophin levels in SLE patients by comparison with RA, Sjögren's syndrome (SS) and healthy controls (HCs), and analyzed its correlations with major clinical features. Due to the relationship between midkine and Th17, we investigated the correlation between the two cytokines and IL-17 in SLE. Furthermore, we also evaluated the potential of midkine and pleiotrophin as SLE biomarkers.

## RESULTS

### The general features of study subjects

The general features of study subjects were summarized in Table 1. There was no significant difference in age and gender distribution between SLE patients and HCs (both *P* > 0.05). The average disease duration of SLE patients was 3.92(0.00, 8.83) years, the mean SLEDAI-2K was 16.64 ± 8.48.

**Table 1:** The general features of study subjects

Parameters	SLE patients (*n*= 83)	Healthy controls (*n* = 82)
Age (year)	36.58 ± 13.41	37.17 ± 13.11
Sex (female/male)	76/7	75/7
Disease duration (year)	3.92 (0.00, 8.83)	NA
SLEDAI-2K	16.64 ± 8.48	NA
Disease manifestations		NA
Renal disease	49 (59)	NA
Vasculitis	6 (7)	NA
Arthritis	40 (48)	NA
Myositis	8 (10)	NA
Rash	43 (52)	NA
Alopecia	37 (45)	NA
Oral ulcer	14 (17)	NA
Pleuritis	6 (7)	NA
Leukopenia	15 (18)	NA
Thrombocytopenia	21 (25)	NA
Fever	29 (35)	NA
Nervous system disorder	18 (22)	NA
Low complement	66 (80)	NA
Autoantibodies		NA
Anti-dsDNA	55 (66)	NA
Anti-Sm	40 (48)	NA
Anti-SSA	58 (70)	NA
Anti-SSB	11 (14)	NA
Anti-RNP	31 (37)	NA
Anti-Ribosomal P	27 (33)	NA
Medical therapy		NA
Prednisone dose ≤ 30 mg/day	43 (52)	NA
Prednisone dose > 30 mg/day	40 (48)	NA
Antimalarials	75 (90)	NA
Azathioprine, MTX, or CTX	13 (16)	NA

Normally distributed data were expressed as means ± standard deviation (SD), whereas variables with a skewed distribution were presented as median (interquartile range).

Categorical variables values are the number (%).

SLE: systemic lupus erythematosus; SLEDAI-2K: systemic lupus erythematosus disease activity Index 2000; dsDNA: double stranded DNA; Sm: Smith; SSA: Sjögren's syndrome-related antigen A; SSB: Sjögren's syndrome-related antigen B; RNP: Ribonucleoprotein; MTX: methotrexate; CTX: cyclophosphamide.

### Comparison of plasma midkine and pleiotrophin levels between SLE patients and HCs, and different subgroups of SLE patients

Both midkine and pleiotrophin levels were significantly increased in the plasma of SLE patients compared with HCs (*P* = 0.018 and *P* = 0.020 respectively). However, no significant differences in midkine and pleiotrophin levels were observed between SLE without nephritis and SLE with nephritis (*P* = 0.774 and *P* = 0.410 respectively). No significant differences in midkine and pleiotrophin levels were observed between less active SLE and more active SLE (*P* = 0.609 and *P* = 0.782 respectively) (Table 2).

**Table 2:** Comparison of plasma midkine and pleiotrophin levels between SLE patients and healthy controls, and different subgroups of SLE patients

Group	Number	Midkine (pg/ml)	Pleiotrophin (pg/ml)
Healthy controls	82	628.22 (373.66, 712.41)	394.37 (231.98, 458.73)
SLE patients	83	698.37 (516.09, 767.07)*	434.82 (332.88, 496.22)*
SLE without nephritis	34	696.29 (533.83, 747.21)	436.57 (376.96, 505.56)
SLE with nephritis	49	707.70 (491.84, 774.30)	404.89 (297.47, 495.45)
Less active SLE	20	690.51 (522.02, 738.93)	441.51 (357.50, 491.20)
More active SLE	63	702.31 (508.46, 773.73)	424.83 (303.29, 498.12)

*versus healthy controls *P* < 0.05.

SLE: systemic lupus erythematosus.

### Comparison of plasma midkine and pleiotrophin levels among SLE patients, RA patients, SS patients and HCs

**Figure 1:**
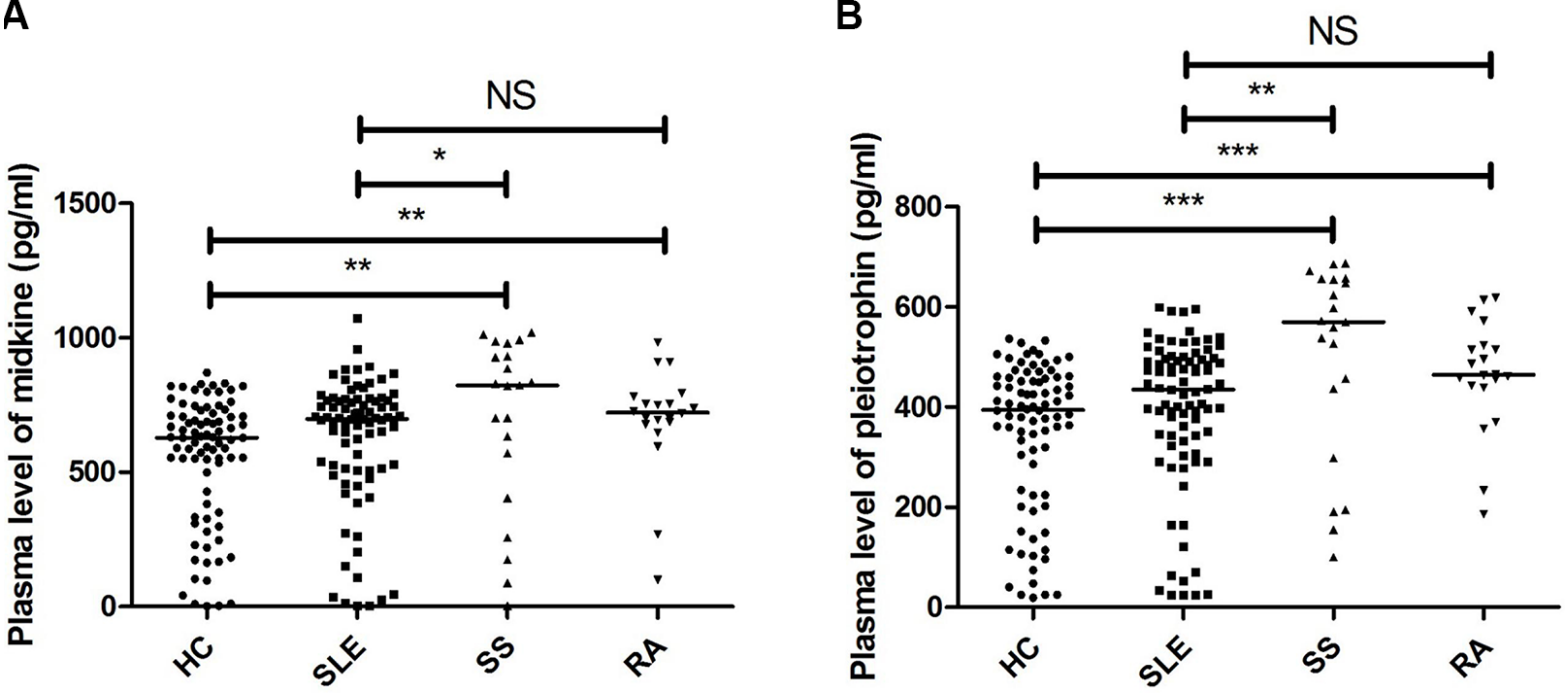
Comparison of plasma midkine and pleiotrophin levels among SLE patients, RA patients, SS patients and healthy controls (**A**) midkine, (**B**) pleiotrophin *P < 0.05, ^*^P < 0.01, ^**^*P < 0.001. NS: not significant.

Significant higher plasma midkine and pleiotrophin levels were observed in RA versus HC (*P* < 0.01 and *P* < 0.001, respectively) and SS versus HC (*P* < 0.01 and *P* < 0.001, respectively). There were significantly lower plasma midkine and pleiotrophin levels in SLE patients compared to SS patients (*P* < 0.05 and *P* < 0.01, respectively). No significant differences in plasma midkine and pleiotrophin levels were found between SLE patients and RA patients (*P* = 0.240 and *P* = 0.074, respectively) (Figure 1).

### Correlations of plasma midkine and pleiotrophin levels with clinical parameters of SLE patients

Associations of plasma midkine and pleiotrophin levels with major clinical parameters of SLE patients were analyzed, and the results showed that both plasma midkine and pleiotrophin levels were associated with rash (*P* < 0.01 and *P* < 0.05, respectively) and anti-SSA (both *P* < 0.01, respectively) in SLE patients. No significant associations of plasma midkine and pleiotrophin levels with other clinical parameters were observed (all *P* > 0.05) (Tables 3, 4).

**Table 3:** Correlations of plasma midkine and pleiotrophin levels with categorical clinical parameters of SLE patients

Characteristic	Number	Midkine	Pleiotrophin
Vasculitis			
Yes	6	731.60 (579.50, 764. 80)	478.86 (320.94, 499.55)
No	77	695.87 (512.27, 773.62)	430.87 (328.07, 497.17)
Arthritis			
Yes	40	703.33 (522.00, 766.15)	420.45 (348.15, 499.57)
No	43	695.21 (506.60, 773.50)	436.15 (307.32, 496.22)
Myositis			
Yes	8	633.30 (61.55, 815.68)	400.88 (35.49, 537.25)
No	75	698.37 (526.99, 767.07)	434.82 (343.32, 496.22)
Rash			
Yes	43	669.25 (458.39, 721.98)^*^	393.44 (290.69, 484.60)*
No	40	740.90 (652.08, 790.81)	475.31 (394.41, 514.60)
Alopecia			
Yes	37	695.87 (440.73, 771.83)	404.89 (297.47, 504.89)
No	46	701.82 (559.73, 768.27)	437.23 (373.95, 491.95)
Serositis			
Yes	6	668.03 (369.88, 785.17)	382.65 (190.12, 468.72)
No	77	698.37 (516.10, 770.29)	436.15 (338.05, 497.17)
Oral ulcer			
Yes	14	695.54 (489.09, 753.01)	414.58 (312.38, 506.55)
No	69	701.33 (512.29, 773.62)	436.15 (338.10, 495.45)
Nervous system disorder			
Yes	18	713.49 (641.15, 776.83)	464.86 (380.72, 498.78)
No	65	695.87 (497.76, 766.80)	430.87 (305.30, 494.39)
Low complement			
Yes	66	701.82 (507.99, 775.30)	432.84 (319.27, 496.20)
No	17	684.30 (563.46, 742.34)	446.88 (354.12, 497.17)
Leukopenia			
Yes	15	680.64 (458.39, 750.50)	406.07 (278.61, 484.60)
No	68	703.33 (519.43, 773.67)	437.23 (343.88, 501.90)
Thrombocytopenia			
Yes	21	695.07 (502.51, 734.68)	397.36 (282.71, 475.31)
No	62	701.82 (522.36, 774.02)	442.60 (340.63, 501.14)
Anti-dsDNA			
Yes	55	696.72 (488.92, 773.73)	424.83 (291.57, 491.46)
No	28	704.52 (581.34, 764.83)	458.26 (366.45, 500.10)
Anti-Sm			
Yes	40	695.14 (498.44, 748.84)	405.48 (301.90, 491.68)
No	43	709.90 (516.09, 777.54)	446.88 (343.32, 512.16)
Anti-SSA			
Yes	58	712.39 (650.02, 776.83)^*^	472.30 (376.91, 509.79)^*^
No	25	539.74 (330.93, 726.62)	377.88 (228.37, 449.15)
Anti-SSB			
Yes	11	704.86 (655.87, 767.07)	471.23 (397.89, 509.01)
No	70	696.29 (485.96, 768.27)	415.45 (303.40, 495.06)
Anti-RNP			
Yes	31	695.21 (529.38, 767.07)	406.07 (343.22, 484.60)
No	52	700.34 (484.93, 772.79)	442.60 (299.56, 501.90)
Anti-Ribosomal P			
Yes	27	695.21 (506.60, 766.53)	406.07 (291.66, 488.79)
No	55	702.31 (516.09, 773.73)	446.88 (343.32, 496.22)
Prednisone dose			
≤ 30 mg/day	43	696.72 (539.74, 764.23)	424.83 (362.64, 496.22)
>30 mg/day	40	704.61 (508.97, 774.53)	435.48 (294.56, 498.71)
Immunosuppressant			
Yes	76	696.29 (508.97, 773.67)	432.84 (311.30, 495.83)
No	7	721.98 (516.12, 746.11)	447.09 (345.59, 498.12)

Immunosuppressant included antimalarials, azathioprine, methotrexate and cyclophosphamide.

SLE: systemic lupus erythematosus; dsDNA: double stranded DNA; Sm: Smith; SSA: Sjögren's syndrome-related antigen A; SSB: Sjögren's syndrome-related antigen B; RNP: Ribonucleoprotein.

**Table 4:** Correlations of plasma midkine and pleiotrophin levels with quantitative clinical parameters of SLE patients

Parameters	*N*	Midkine		Pleiotrophin	
		*r*_s_	*P* value	*r*_s_	*P* value
C3	83	0.099	0.375	0.160	0.149
C4	80	0.008	0.945	0.046	0.688
ESR	81	0.108	0.337	0.105	0.351
CRP	81	0.041	0.719	0.044	0.697
SLEDAI-2K	83	0.028	0.803	−0.068	0.544
duration	83	0.102	0.358	0.130	0.241

SLE: systemic lupus erythematosus; SLEDAI-2K: systemic lupus erythematosus disease activity index 2000; ESR: Erythrocyte Sedimentation Rate; CRP: C Reactive Protein; C3: Complement 3; C4: Complement 4.

### Correlations of plasma midkine and pleiotrophin levels with IL-17 level

**Figure 2:**
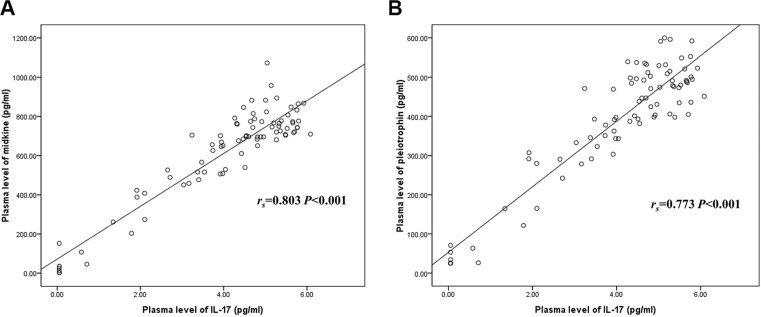
Correlations of plasma midkine and pleiotrophin levels with IL-17 level (**A**) Correlation of midkine with IL-17, (**B**) Correlation of pleiotrophin with IL-17. IL-17: interleukin 17.

Both midkine and pleiotrophin levels were positively correlated with IL-17 level (*r*_s_ = 0.803 *P* < 0.001, *r*_s_= 0.773 *P* < 0.001, respectively) (Figure 2).

### Evaluation on diagnostic performance of midkine and pleiotrophin as SLE biomarkers

**Figure 3:**
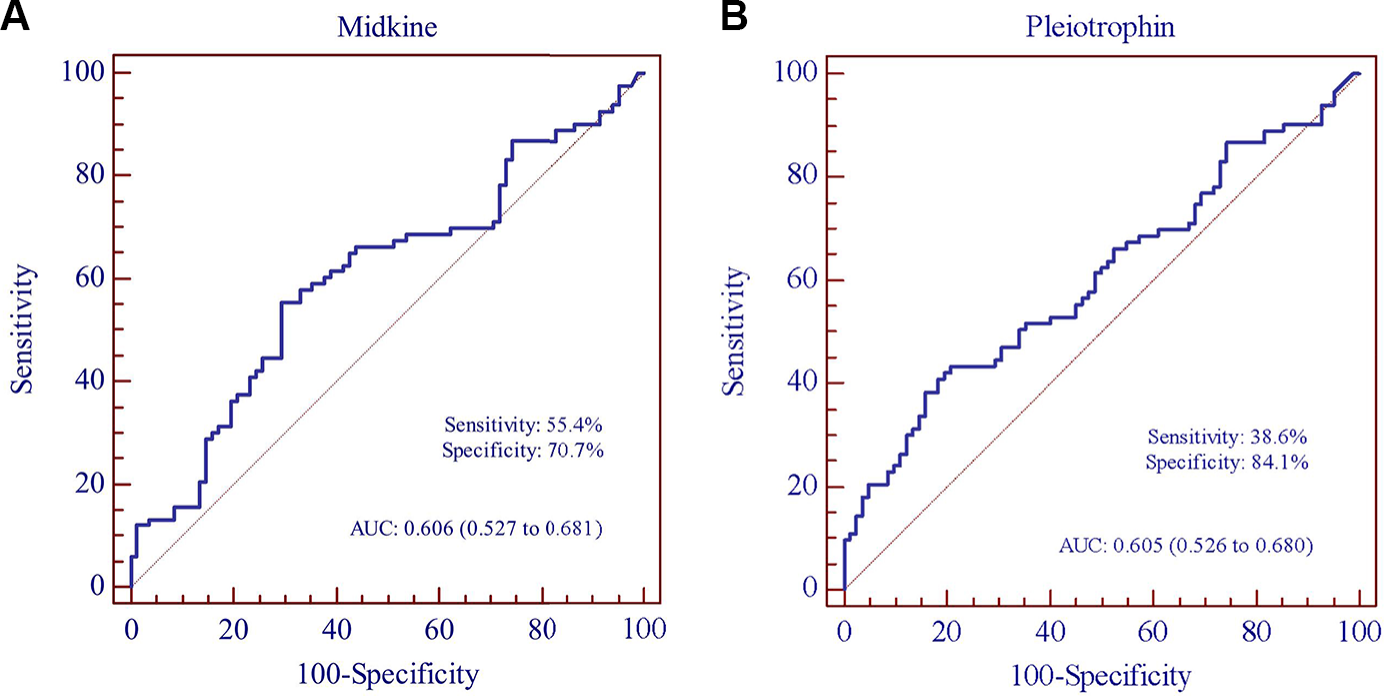
Receiver operating characteristic (ROC) curve analysis of midkine and pleiotrophin for the discriminative ability of SLE patients versus healthy controls (**A**) midkine, (**B**) pleiotrophin.

The AUC of the ROC curve for midkine and pleiotrophin were 0.606 (0.527–0.681) and 0.605 (0.526–0.680) respectively (Figure 3).

## DISCUSSION

This study for the first time examined plasma midkine and pleiotrophin expression in SLE patients. Our results are similar to the previous relevant studies in other autoimmune diseases [17, 21], such as elevated plasma midkine level and its association with IL-17 in SLE patients, and elevated plasma midkine level in RA. We provide novel evidence that plasma midkine and pleiotrophin levels are increased in SS. However, we did not find association between midkine levels and SLE disease activity. In addition, we also evaluated the potential of midkine and pleiotrophin as SLE biomarkers. However, the results did not indicate a better diagnostic value usable in the clinical practice.

In recent years, there is growing evidence that midkine are implicated in autoimmune diseases. In RA patients, midkine levels in serum or synovial fluid were elevated [13, 15], and its levels were positively correlated with IL-17 and C-reactive protein (CRP), but not with Disease Activity Score (DAS), rheumatoid factor, erythrocyte sedimentation rate (ESR), anti-keratin antibodies and anti-cyclic citrullinated peptide antibodies [21]. In antibody-induced arthritis, mice deficient in *midkine* gene (Mdk(−/−) mice) rarely developed the disease, while most of the wild-type mice did. Administration of midkine to Mdk(−/−) mice increased the disease risk, migration of inflammatory leukocytes to the synovial membranes in the disease model was inhibited in Mdk(−/−) mice. Moreover, midkine was found to promote the differentiation of osteoclasts from macrophages [13]. A chimeric-type siRNA for midkine suppressed the development of antibody-induced arthritis and adhesion of the omentum to the injured abdominal wall [14]. More recently, Shindo *et al*. reported that elevated serum midkine level was associated with higher DAS28-ESR, disability index of the Health Assessment Questionnaire, and rheumatoid factor level. Anti-TNF therapy significantly downregulated the serum midkine level. Midkine was expressed by synovial lining cells in RA synovial tissues and it upregulated the production of IL-6, IL-8, and CCL2 by rheumatoid synovial fibroblasts [15]. Therefore, midkine may be implicated in the pathogenesis of RA via induction of inflammatory mediators. Takeuchi *et al*. showed that removal of midkine suppressed EAE due to the expansion of the Treg cell population and decrease in the numbers of autoreactive Th1 and Th17 cells. Midkine decreased Treg cell population by inhibiting the STAT5 phosphorylation, which is essential for the Foxp3 expression. Furthermore, midkine reduced DCreg cell population through suppressing the STAT3 phosphorylation, which is crucial for DCreg development. Blockade of midkine signaling with a specific RNA aptamer significantly elevated the population of DCreg and Treg cells and ameliorated EAE without detectable adverse effects [16, 18]. Inflammatory bowel diseases (IBDs), including ulcerative colitis (UC) and Crohn's disease (CD), are systemic, chronic inflammatory diseases. In CD, serum midkine levels was positively associated with Crohn's Disease Activity Index, and midkine was found to be a sensitive biomarker of diagnostic value comparable with the gold standard CRP in this disease [20]. In an experimental colitis model, a well-established model for UC, midkine was abundantly expressed in fibroblasts of the mucosal and submucosal layers of the rat distal colon [25]. In UC, midkine was higher in inactive and active UC compared with controls, and positively associated with disease activity. Midkine also corresponded with clinical, endoscopic, inflammatory and angiogenic activity, and anemia. Performance of midkine as a marker of UC or active UC was comparable to that of CRP [19]. Collectively, these evidence suggest that midkine may play a key role in the pathogenesis of autoimmune diseases including SLE, inhibiting midkine therefore might be useful for attenuating inflammation-related symptoms in these diseases.

Despite increasing evidence of the involvement of midkine in the pathogenesis of autoimmune diseases, the data on pleiotrophin in these diseases are very limited. An earlier study showed that the basal expression of pleiotrophin mRNAs in normal spinal cords was significantly upregulated after induction of EAE, and its expression reached peak levels threefold above basal levels during the clinical recovery period, suggesting its role in the disease progression [26]. Using MRL-lpr/lpr mice, a mouse model for SLE, Asari *et al*. showed that that oral administration of high molecular weight hyaluronan (HA900) inhibited Th1-type autoimmune disease and inflammation by up-regulating SOCS3 expression and down-regulating pleiotrophin expression [27]. In human SLE, *pleiotrophin* gene single-nucleotide polymorphism (SNP) [rs919581] was confirmed to be associated with susceptibility to SLE [28]. All the available evidence indicate that pleiotrophin may also be involved in SLE.

Several limitations in this study should be acknowledged. First, this study is limited by a small sample size that presented to two tertiary Hospitals, which may restrict the generalizability of our results. Second, this is a cross-sectional study, the exact mechanism of these two cytokines in the development and pathogenesis of SLE was not explored, which also makes determining a causal relationship between midkine and SLE challenging. Therefore, further studies are still required to determine the exact role of midkine in SLE pathogenesis.

Taken together, elevated plasma midkine and pleiotrophin levels and their associations with rash, anti- SSA and IL-17 in SLE patients suggest their important role in this disease.

## MATERIALS AND METHODS

### Study subjects

83 SLE patients and 123 controls including 20 RA patients, 21 SS patients and 82 community based HCs were recruited. SLE diagnosis was made by the presence of four or more 1997 revised American College of Rheumatology (ACR) classification criteria. Renal involvement of SLE patients was determined according to the ACR criteria, i e, any one of the following: 1) persistent proteinuria ≥ 0.5 g/day; 2) the presence of active cellular casts; or 3) biopsy evidence of lupus nephritis. The disease severity was quantified according to the Systemic Lupus Erythematosus Disease Activity Index 2000 (SLEDAI-2K) [29]. Disease activity was quantified using the SLEDAI-2K score. More active SLE was defined as a SLEDAI-2K score > 10, those patients with SLEDAI-2K ≤ 10 were classed as relatively inactive [30, 31]. The patients with RA were diagnosed according to the ACR/European League Against Rheumatism 2010 classification criteria [32]. The patients with SS were diagnosed in accordance with the revised 2002 American-European criteria [33]. Exclusion criteria of all patients were as follows: (i) patients with malignant tumors; (ii) patients with serious acute infection within six weeks before admission; (iii) patients complicated with other autoimmune disease; and (iv) patients suspected of drug or alcohol abuse. Demographic and clinical data were collected from hospital records or by questionnaire. The study was approved by the Medical Ethics Committee of Anhui Medical University. Methods were carried out in accordance with the approved guidelines. All subjects were enrolled after informed consent had been obtained.

### Extraction of plasma and enzyme-linked immunosorbent assay (ELISA)

Plasma were obtained from 5 ml of whole blood of all study subjects and stored at −80°C until use. Plasma midkine, pleiotrophin and IL-17 levels were detected by specific ELISA kits according to the manufacturer's recommendation (R&D Systems, Inc.), the results were expressed as picograms per milliliter.

### Statistical analysis

Numerical data were expressed as mean ± SD, or median (interquartile range, IQR) if they were not in normal distribution. Difference of continuous variables in different groups was compared with *t* test or Mann–Whitney rank sum test. The Chi-square test was used to assess differences in categorical data between two groups. Spearman's rank correlation coefficient was used for the correlation analysis. Receiver operating characteristic (ROC) curves were constructed and the area under curve (AUC) was used to assess specificity and sensitivity of predictive power or feasibility of using plasma midkine and pleiotrophin as biomarkers for SLE. All statistical analysis was conducted using the Statistical Package for the Social Sciences (SPSS) statistical software for Windows, Version 10.01 (SPSS Inc, IL, USA). The ROC curve analyses were performed with MedCalc version 11.4.2.0 (Mariakerke, Belgium). *P* < 0.05 was considered as statistically significant.
